# The frozen elephant trunk technique in acute aortic dissection: the ultimate solution? An institutional experience

**DOI:** 10.3389/fcvm.2024.1330033

**Published:** 2024-07-30

**Authors:** K. Wisniewski, A. M. Dell’Aquila, A. Motekallemi, A. Oberhuber, J. F. Schäfers, E. Marchiori, R. Weber, S. Martens, A. Rukosujew

**Affiliations:** ^1^ Department of Cardiothoracic Surgery, University Hospital Muenster, Muenster, Germany; ^2^ Department of Cardiac Surgery, University Hospital Halle (Saale), Halle (Saale), Germany; ^3^ Department of Cardiac Surgery, Klinikum Kassel, Kassel, Germany; ^4^ Department of Vascular and Endovascular Surgery, University Hospital Muenster, Muenster, Germany

**Keywords:** Thoraflex hybrid prosthesis, acute aortic dissection, aortic arch repair, hemiarch replacement, frozen elephant trunk (FET)

## Abstract

**Objective:**

Acute aortic dissection remains a serious emergency in the field of cardiovascular medicine and a challenge for cardiothoracic surgeons. In the present study, we seek to compare the outcomes of different surgical techniques in the repair of type A acute aortic dissection.

**Methods:**

Between April 2015 and May 2023, 213 patients (82 women, aged: 63.9 ± 13.3 years) with acute aortic dissection (205 type A and 8 non-A-non-B dissections) underwent surgical treatment in our department. A total of 45 patients were treated with the frozen elephant trunk (FET) technique supported by the Thoraflex™ Hybrid prosthesis, 33 received total aortic arch replacement (TAR)—standard or conventional elephant trunk—treatment, and 135 were treated with hemiarch replacement (HR). Aortic arch surgery was performed in most patients under moderate hypothermic (28°C on average) circulatory arrest, with selective antegrade cerebral perfusion through the right axillary artery.

**Results:**

The rates of early mortality were 17.8% (38 perioperative deaths) in the whole population, 8.9% in the FET group of patients, and 33% and 17% in the TAR and HR group of patients, respectively (*P*-value 0.025). The rate of spinal cord injury was 2.3% (five patients), and a paresis of recurrent laryngeal occurred in 3.7% of patients (seven patients, four were treated with FET). Permanent neurological dysfunction occurred in 27 patients (12.7%). After a mean follow-up of 3 years, the rate of mid-term mortality of discharged patients was 19.4% (34 deaths: 7 FET, 4 TAR, and 23 HR) and the overall mortality rate was 33.8% [72 deaths: 11 FET (24.4%); 15 TAR (45.4%); 46 HR (34.1%)]. A total of 8 patients (17.8%) in whom FET was applied received additional endovascular treatment in the descending aorta.

**Conclusions:**

In our institutional experience, we found that the frozen elephant trunk technique with a high-end Thoraflex Hybrid prosthesis proved its surgical suitability in the treatment of acute aortic dissection with favorable outcomes. The FET technique and our perioperative management led to comparable neurological outcomes and reduced mortality rates in these emergency cases.

## Introduction

**Figure 1:**
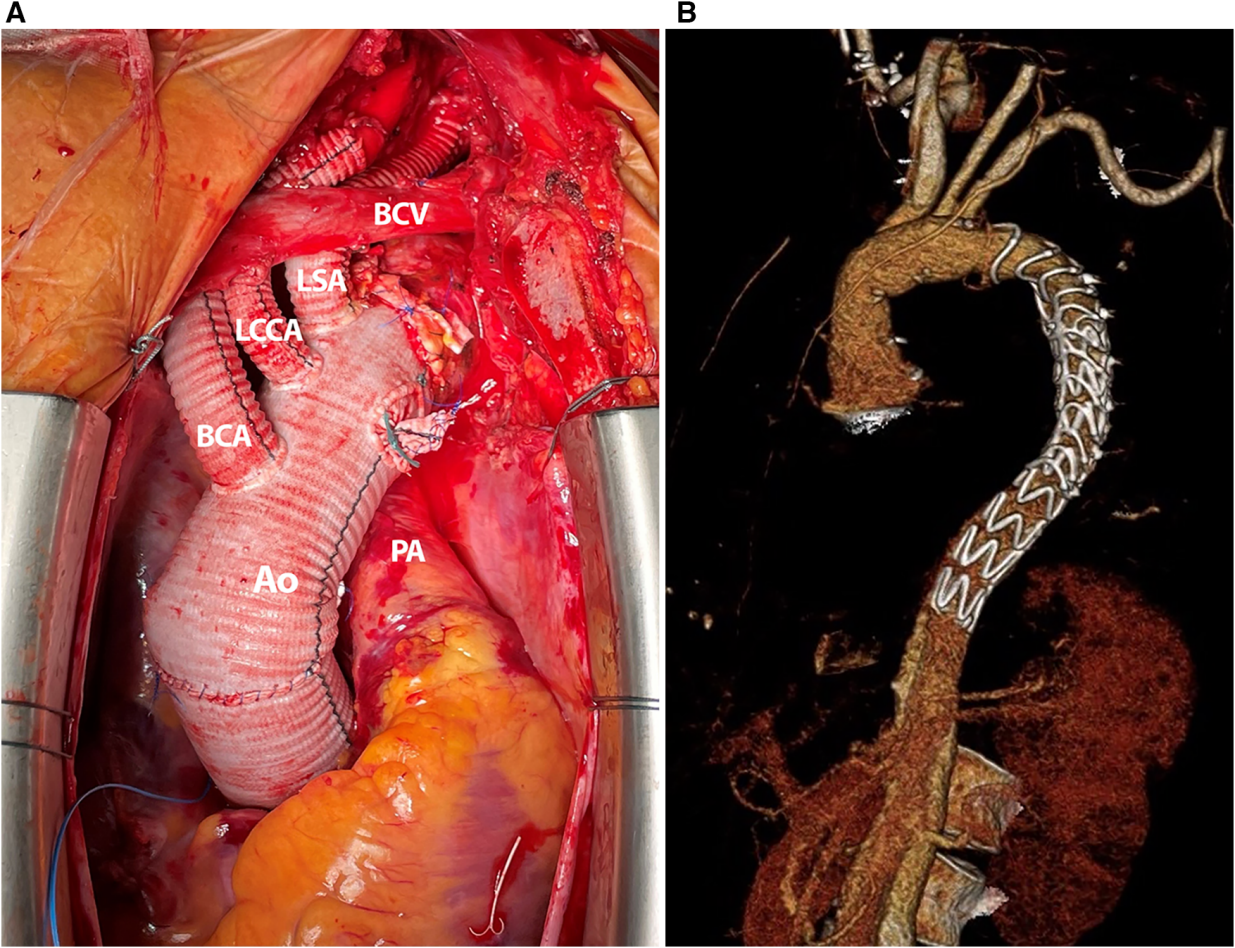
(**A**) Intraoperative photography, complete implanted Thoraflex hybrid prosthesis with extensive left subclavian artery repair due to dissection. (**B**) A 3D CT reconstruction after frozen elephant trunk repair with Thoraflex™ Hybrid prosthesis and distal stent graft extension.

Since the introduction of the frozen elephant trunk (FET) technique, aortic arch surgery has undergone a kind of “renaissance” with improved management of complex vascular conditions. With the implementation of hybrid prostheses such as E-Vita® Open (JOTEC GmbH, Hechingen, Germany/Artivion, Inc., Kennesaw, Georgia, USA) or Thoraflex™ Hybrid (Vascutek, Terumo, Inchinnan, Scotland, UK) (1) among others, the surgical community has received irreplaceable tools in their armamentarium (Figures 1A,B). This type of surgery has become a widely established approach for the treatment of elective aortic diseases such as aneurysms of the thoracic aorta as well as postdissection aneurysms, and acute and chronic aortic dissections, including Type A, B, and non-A non-B dissections (nAnB) (2–5).

However, it still needs to be clarified how the treatment of thoracic aortic pathologies involving the aortic arch, such as acute aortic dissection, should be performed (2). Valuable and useful insights have provided us with new guidelines for treating the pathologies of the aortic organ. The standardization of therapy regarding the location of tear entry has simplified the decision-making process (6). Meanwhile, there is an ongoing discussion on whether one should “freeze” all the dissections with FET to sustain a positive effect on remodeling in the downstream of the aorta and possible subsequent endoluminal treatment, as previously described (7, 8). Alternatively, should we be more conservative and restrictive in handling patients with complex conditions, as in the elderly population with many comorbidities, to shorten operation time, and do the “necessary minimum”? In light of such dilemmas, we present our institutional experience regarding the management of acute aortic dissection and the place occupied by Thoraflex™ Hybrid in our clinical setting.

## Materials and methods

### Patients

**Figure 2:**
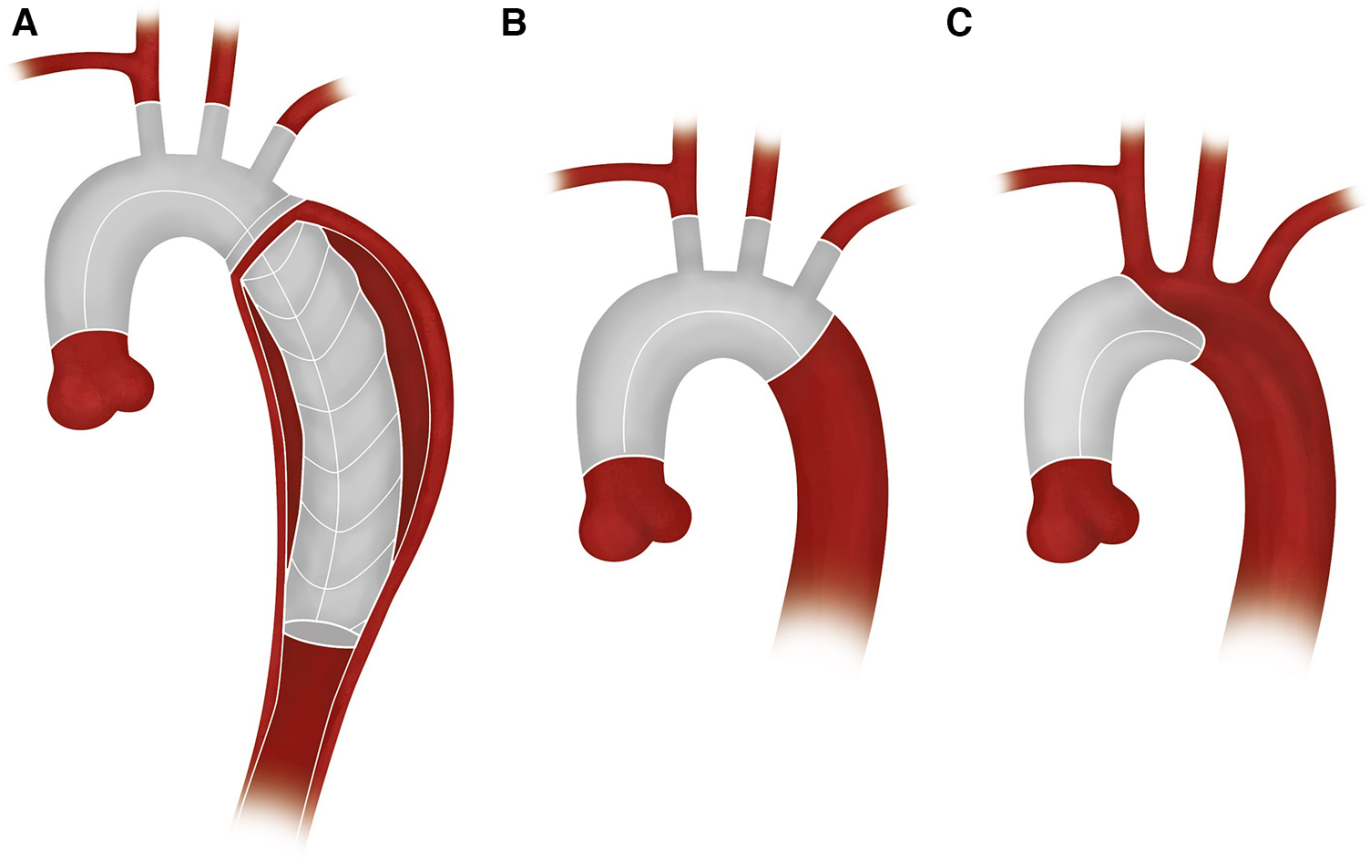
Schematic diagrams: (**A**) FET, (**B**) TAR, and (**C**) HR.

Between April 2015 and May 2023, 213 patients (82 women, 63.9 ± 13.3 years) with acute aortic dissection (205 type A—AADA and 8 nAnB) underwent surgical treatment at our department. A total of 45 patients were treated with the frozen elephant trunk technique with the Thoraflex™ Hybrid prosthesis (Terumo Aortic, Vascutek Ltd., Inchinnan, UK), 33 received total aortic arch replacement (TAR)—standard or conventional elephant trunk (CET)—and 135 were treated with hemiarch replacement (HR). Schematic diagrams are shown in Figure 2. This retrospective study was approved by the ethical committee of our institution (2020-126-f-S). The perioperative patient data were reviewed retrospectively. The follow-up data regarding survival were retrieved from the registration offices of the residents. In September 2023, follow-up was completed. The data were analyzed according to the underlying operation technique (FET, TAR, or HR). Patient demographics are presented in Table 1. The diagnosis of acute aortic dissection was preferably confirmed by electrocardiogram (ECG)-gated computed tomography angiography (CTA). However, in many patients, surgery was initiated based on the findings of standard CTA and confirmed echocardiography results. The same surgical team performed all FET procedures. For the total aortic arch replacement or hemiarch replacement procedure, all attending surgeons of our department were involved. The surgical strategy was chosen based on the experience and preference of the surgeons.

**Table 1:** Demographic and preoperative clinical data.

	All *N* = 213 [*n*/mean (%/±SD)]	FET, *N* = 45 [*n*/mean (%/±SD)]	Non-FET arch repair, *N* = 33 [*n*/mean (%/±SD)]	Hemiarch, *N* = 135 [*n*/mean (%/±SD)]	*P*-value
Age (years)	63.9 (±13.3)	57.1 (±10.0)	61.8 (±13.7)	66.7 (±13.3)	**<0**.**001**
Sex (female)	82 (38.5%)	8 (17.8%)	10 (30.3%)	64 (47.4%)	**0**.**006**
Body mass index	27.6 (±5.3)	26.6 (±4.3)	27.8 (±4.3)	27.9 (±5.8)	0.534
History of smoking	54 (25.3%)	10 (22.2%)	10 (30.3%)	34 (25.2%)	0.792
Diabetes	10 (4.7%)	1 (2.2%)	1 (3.0%)	8 (5.9%)	0.613
Dyslipidemia	43 (20.2%)	9 (20.0%)	5 (15.1%)	29 (21.5%)	0.426
Arterial hypertension	144 (67.6%)	29 (64.4%)	22 (66.7%)	93 (68.9%)	0.796
Peripheral vascular disease	3 (1.4%)	1 (2.2%)	0 (0%)	2 (1.5%)	0.588
Cerebrovascular disease	6 (2.8%)	2 (4.4%)	0 (0%)	4 (3.0%)	0.687
Abdominal aortic aneurysm	5 (2.3%)	2 (4.4%)	1 (3.0%)	2 (1.5%)	0.67
Pulmonary disease	18 (8.4%)	3 (6.7%)	2 (6.1%)	13 (9.6%)	0.908
Preoperative dialysis	3 (1.4%)	0 (0%)	0 (0%)	3 (2.2%)	0.709
Preoperative atrial fibrillation	25 (11.7%)	5 (11.1%)	3 (9.1%)	17 (12.6%)	0.66
Preoperative syncope	21 (9.8%)	7 (15.5%)	2 (6.1%)	12 (8.9%)	0.159
Preoperative CPR	8 (3.7%)	2 (4.4%)	1 (3.0%)	5 (3.7%)	0.238
Preoperative catecholamines	27 (12.7%)	4 (8.9%)	3 (9.1%)	20 (14.8%)	0.596
Preoperative ventilation	13 (6.1%)	1 (2.2%)	1 (3.0%)	11 (8.1%)	0.326
Preoperative new-onset cerebral ischemia or paraplegia	41 (19.2%)	12 (26.7%)	4 (12.1%)	25 (18.5%)	0.183
Preoperative cardiac ischemia	6 (2.8%)	2 (4.4%)	1 (3.03%)	3 (2.2%)	0.615
Previous thoracoabdominal aortic repair	7 (3.3%)	0 (0%)	3 (9.1%)	4 (3.0%)	0.097
GERAADA score (%)	22.2 (±12.8)	24.8 (±9.9)	20.3 (±13.6)	21.8 (±13.4)	**0**.**008**

Values in bold are statistically relevant.

The presence of preoperative comorbidities such as history of smoking, diabetes, abnormally high amounts of any lipids or lipoproteins in the blood, arterial hypertension, any peripheral vascular disease, relevant obstruction of the cerebral circulation, abdominal aneurysms, any pulmonary disease, kidney failure requiring dialysis, genetic syndromes, and atrial fibrillation was noted.

Preoperative malperfusion was characterized according to imaging findings and clinical signs of end-organ malperfusion. Cardiac ischemia was defined as coronary artery dissection with or without clinical signs/symptoms typical of myocardial malperfusion. The dissection of at least one supra-aortic vessel with or without clinical symptoms of cerebral malperfusion (stroke) was defined as preoperative new-onset cerebral ischemia. The dissection or false lumen of the iliac artery, with or without clinical symptoms of lower-extremity ischemia, was recorded. Preoperative resuscitation, loss of consciousness, necessity of ventilation, or use of catecholamines was also documented.

### Surgical technique and perioperative management

All patients received the same standardized perioperative management as described previously (9). The primary surgical setting for all acute dissection cases remained the same, regardless of the team member who performed the aortic repair (median sternotomy with an incision in the deltopectoral groove), but customarily, the approach needed to be adjusted according to perioperative findings, e.g., extension of sternotomy or additional incision to expose the femoral vessels. Arterial cannulation was performed directly through the right axillary artery and venous cannulation through the right atrium. In some patients, due to anatomical reasons or local direct aortic dissection, cannulation was performed on or through the femoral vessels. The patients’ blood pressure levels were monitored with a standardized placement of three arterial lines in the bilateral radial arteries and in the femoral artery (if it itself was not affected by dissection). The venting of the left side of the heart was performed through the right superior pulmonary vein. Intermittent cold blood cardioplegia was administered in most retrograde patients through the coronary sinus or directly into the coronary ostia. The cardioplegia infusion was repeated every 20 min. The aortic arch surgery was performed under moderate hypothermic (28°C on average) circulatory arrest (except in two patients with deep hypothermia), with selective antegrade cerebral perfusion (SACP) and the application of near-infrared spectroscopy (NIRS). If necessary, the unilateral perfusion could be switched to a bilateral one through direct cannulation of the left common carotid artery.

The hemiarch replacement procedure was executed in the proximal part of the arch beyond the level of the innominate artery, without involving the arch vessels. HR was performed using an open technique with moderate hypothermia and selective antegrade cerebral perfusion.

In the SACP and Trendelenburg position, the aortic cross-clamp was released, and the final inspection of the aortic arch was conducted to investigate the severity of dissection and to identify the entry. Afterward, the final decision was made on the type of repair to be performed. The surgical principles of total arch repair are similar to those of FET, as described previously (9).

After completion of the distal anastomosis, lower body reperfusion was obtained via the extracorporeal circulation branch of the graft. To minimize the risk of cardiac arrest, perfusion of the heart was re-established after repairing the subclavian artery and proximal anastomosis of the ascending aorta (in the case of supracoronary replacement). The remaining vessels were then anastomosed (9).

Postoperatively, all patients received CTA before discharge. In those with preoperative signs of malperfusion, CTA was performed directly after surgery to assess possible pathologies and to address them when needed. During follow-up, CTAs were performed 3 months after discharge and thereafter predominantly on a yearly basis, depending on the condition of the patients.

### Statistical analysis

The statistical analysis was performed using R software version 4.2.1 (Foundation for Statistical Computing, Vienna, Austria) and IBM SPSS Statistics for Windows (Version 28.0; IBM Corp, Armonk, NY, USA). Continuous variables were expressed as mean ± standard deviation. Categorical variables were given as total number and percentages. A chi-square (*Χ*^2^) test was used to analyze the differences between dichotomous variables. When normal distribution was confirmed, a parametric test (one-way ANOVA) was used; otherwise, a non-parametric Kruskal–Wallis test was chosen. The Kaplan–Meier survival estimate was used for the survival analysis. The statistical differences in the Kaplan–Meier survival estimate were determined by using the log-rank test. *P*-values ≤0.05 were considered significant. Univariate and multivariate analysis, using a Cox regression model, was performed to identify the preoperative, intraoperative, and postoperative independent predictors of overall mortality. The model was established for factors with univariate *P*-values <0.05 and with multivariate *P*-values <0.2. The results were documented as an odds ratio (OR) with a 95% confidence interval (CI).

## Results

### Perioperative results

The intraoperative and postoperative data are presented in detail in Tables 2, 3 respectively. Overall, 213 patients with the high-operative risk acute aortic dissections (205 type A—AADA and 8 nAnB) were treated with either of the following techniques: frozen elephant trunk, total arch replacement, or hemiarch. Arterial cannulation through the right axillary artery was performed in 89.7% of the patients (191). In the investigated cohort, hemiarch patients were considerably older (*P* < 0.001) than their frozen elephant trunk or the total arch replacement counterparts. In all subgroups, the patients were predominantly men, but the hemiarch cohort was well-balanced in gender fractions. Hemiarch patients had shorter operation times with a statistically significant shorter hypothermic circulatory arrest (HCA) but were more often hemodynamically unstable, which necessitated catecholamine therapy and intubation. Contrastingly, the aortic arch replacement procedure, either FET or CET, lasted longer (FET 338.8 min, CET 374.2 min, HR 304.1 min) but without having any statistical significance.

**Table 2:** Intraoperative data.

	All *N* = 213 [*n*/mean (%/±SD)]	FET, *N* = 45 [*n*/mean (%/±SD)]	Non-FET arch repair, *N* = 33 [*n*/mean (%/±SD)]	Hemiarch, *N* = 135 [*n*/mean (%/±SD)]	*P*-value
Duration of surgery (min)	322.3 (±80)	338.8 (±64)	374.2 (±96)	304.1 (±74)	0.151
CPB time (min)	191.9 (±55)	216.3 (±43)	237.4 (±49)	172.7 (±51)	0.189
Cross-clamp time (min)	118.7 (±46)	132.0 (±57)	151.3 (±48)	106.3 (±36.1)	0.164
Distal HCA time (min)	43.4 (±18)	49.9 (±10)	58.2 (±23)	37.6 (±16)	**0**.**031**
HCA temperature (°C)	28.9 (±2)	28.0 (±1)	28.4 (±2)	29.4 (±2)	**<0.001**
Axillary artery cannulation	191 (89.7%)	40 (88.9%)	30 (91.0%)	121 (89.6%)	0.973
Aortic cannulation	17 (7.9%)	4 (8.9%)	2 (6.1%)	11 (8.1%)	0.9220
Femoral artery cannulation	6 (2.8%)	1 (2.2%)	1 (3.0%)	4 (3.0%)	0.993
Bentall with a biological conduit	17 (7.9%)	6 (13.3%)	2 (6.1%)	9 (6.7%)	0.173
Bentall with a mechanical conduit	20 (9.4%)	4 (8.9%)	4 (12.1%)	12 (8.9%)	0.838
1/3 Yacoub remodeling	9 (4.2%)	2 (4.4%)	4 (12.1%)	3 (2.2%)	**0**.**042**
Additional CABG	13 (6.1%)	1 (2.2%)	3 (9.1%)	9 (6.7%)	0.514
DeBakey I	196 (92.0%)	36 (80.0%)	33 (100%)	127 (94.1%)	**0**.**006**
DeBakey II	9 (4.2%)	1 (2.2%)	0 (0%)	8 (5.9%)	0.277
Non-A-non-B	8 (3.7%)	8 (17.8%)	0 (0%)	0 (0%)	**<0.0001**

Values in bold are statistically relevant.

**Table 3:** Postoperative data.

	All *N* = 213 [*n*/mean (%/±SD)]	FET, *N* = 45 [*n*/mean (%/±SD)]	Non-FET arch repair, *N* = 33 [*n*/mean (%/±SD)]	Hemiarch, *N* = 135 [*n*/mean (%/±SD)]	*P*-value
30-day mortality	38 (17.8%)	4 (8.9%)	11 (33.3%)	23 (17.0%)	**0**.**025**
Length of ICU stay (days)	8.3 (±8.3)	8.2 (±7.1)	7.1 (±9.0)	8.7 (±8.6)	0.576
Length of stay (days)	14.9 (±9.8)	18.1 (±8.6)	13.7 (±11.1)	14.1 (±9.7)	**0**.**052**
Tracheostomy	19 (8.9%)	7 (15.5%)	2 (6.1%)	10 (7.4%)	0.092
Reintubation	28 (13.1%)	6 (13.3%)	7 (21.1%)	15 (11.1%)	0.287
Ventilation time (h)	39.3 (±75.3)	51.8 (±98.4)	32.3 (±49.5)	36.9 (±71.7)	0.093
Re-exploration for bleeding	23 (10.8%)	8 (17.8%)	6 (18.2%)	9 (6.7%)	**0**.**017**
ECLS	12 (5.6%)	0 (0%)	5 (15.1%)	7 (5.2%)	**0**.**021**
Postoperative CPR	10 (4.7%)	3 (6.7%)	4 (12.1%)	3 (2.2%)	0.183
Myocardial infarction	5 (2.3%)	0 (0%)	0 (0%)	5 (3.7%)	0.26
Postoperative stroke (permanent neurological deficit)	27 (12.7%)	5 (11.1%)	5 (15.1%)	17 (12.6%)	0.927
New postoperative paraparesis	5 (2.3%)	1 (2.2%)	0 (0%)	4 (3.0%)	0.609
Recurrent nerve palsy	8 (3.7%)	5 (11.1%)	2 (6.1%)	1 (1.0%)	**0**.**009**
Mesenteric ischemia	5 (2.3%)	1 (2.2%)	1 (14.3%)	3 (2.2%)	0.96
Temporary hemofiltration	49 (23.0%)	12 (26.7%)	10 (30.3%)	27 (20.0%)	0.211
Sternal wound infection	3 (1.4%)	1 (2.2%)	1 (3.0%)	1 (1.0%)	0.492

ICU, intensive care unit.

Values in bold are statistically relevant.

In the FET subgroup, distal anastomosis was performed in 68.9% of patients (31 patients) in zone 3 (distal to the left subclavian artery) and only 31.1% (14 patients) in zone 2. In a few early cases, a retrograde wire was placed in the femoral artery to guide the FET stent placement procedure. However, over the course of time, we found that this maneuver was not obligatory for successful deployment. Subsequent deployment control of Thoraflex was not performed, neither was it essential, as borne out by our experience.

In 16 patients, FET with a 150 mm stent graft (35%) was implanted. The size and the length of the stent graft were determined preoperatively, together with a local vascular surgeon, to create a landing zone of sufficient size for subsequent intervention, if needed.

The mean hospital stay in the cohort lasted 14.9 days. The FET patients had a prolonged ventilation time and an overall longer hospital stay of 18.1 days (*P* = 0.052) compared with all other groups.

Overall, 49 patients (23.0%) required temporary hemofiltration due to acute kidney injury, as also 30.3% patients in the non-FET group. Only seven patients required permanent dialysis after discharge, five of whom were treated with hemiarch replacement.

Five patients (2.3%) suffered from paraparesis (one treated with FET, length of stent 150 mm, and four with HR) and eight (3.7%) from a paresis of the recurrent laryngeal nerve, of whom five were FET patients (11.1%), two were treated with TAR, and one with HR. Postoperative stroke occurred in 27 patients (12.7%) with a similar distribution pattern in all subgroups (11,1%, 15,1%, and 12.6%—FET, TAR, and HR, respectively). In every subgroup, one case of wound infection was reported. We observed five patients with myocardial infarction and mesenteric ischemia as well. Cardiopulmonary reanimation was required in 10 patients (4.7%), of whom four (12.1%) were patients with TAR. Extracorporeal life support (ECLS) was necessary in 12 patients (5.6%), while patients with TAR (5, 15.1%) received significant support more often than the other subgroups. A surgical re-exploration due to bleeding was required in 23 patients (10.8%). To stop bleeding in eight (17.8%) and six (18.2%) patients with FET and TAR, respectively, a reoperation had to be performed. It should be noted, however, that half of the re-exploration surgeries in the FET group were performed in the first 2 years after the introduction of this technique at our institution.

Our cohort represents a high-risk population, with multiple patients suffering from complicated end-organ ischemia. At the time of admission, 41 patients (19.2%) had new-onset cerebral ischemia or paraplegia, of whom the most affected were FET patients [12 cases (26.7%)] and HR patients [25 cases (18.5%)]. This complication is likely to affect the rate of mortality.

The retrospectively calculated GERAADA score (predicted 30-day mortality in patients undergoing surgery for acute type A aortic dissection) of the whole cohort was 22.2%, which reflects the high risk of this population. The predicted 30-day rates of mortality were 24.8% in the FET group, 20.3% in the TAR group, and 21.8% in the HR group.

### In-patient mortality

The early mortality rate expressed as 30-day mortality was 17.8% (38 perioperative deaths) in the whole population, where the differences were statistically significant (a log-rank *P*-value of 0.025 in favor of FET patients). In patients with acute aortic dissection treated with TAR, 11 died, translating into a relatively high mortality rate of 33.3% (due to multiple-organ failures, mesenteric or cerebral ischemia, cerebral bleeding; 5 of them died while supported by ECLS). After HR, we observed 23 deaths (17% due to multiple-organ failures and mesenterial or cerebral ischemia; 6 of them died on ECLS support). In contrast, four patients (8.9%) treated with FET died in the early postoperative course.

Among perioperative variables, in the univariate analysis, age, preoperative syncope, preoperative ventilation at admission, duration of surgery, cardiopulmonary bypass (CBP) time, additional coronary artery bypass graft (CABG) during surgery, overall length of hospitalization, ventilation time, ECLS support, postoperative cardiopulmonary resuscitation (CPR), postoperative myocardial infarction, stroke, mesenteric ischemia, and temporary hemofiltration were all associated with overall mortality. In the multivariate analysis, age (*P* = 0.008, OR 1.034, 95% CI 1.009–1.060), preoperative ventilation at admission (*P* = 0.008, OR 3.142, 95% CI 1.357–7.279), duration of surgery (*P* = 0.021, OR 1.004, 95% CI 0.999–1.010), length of hospitalization (*P* < 0,001, OR 0.899, 95% CI 0.856–0.944), ECLS support (*P* = 0.002, OR 6.486, 95% CI 1.945–21.623), stroke (*P* < 0.001, OR 3.335, 95% CI 1.653–0.728), and temporary hemofiltration (*P* = 0.014, OR 2.279, 95% CI1.181–4.398) were identified as independent risk factors for mortality*.* However, the use of different surgical approaches was not found to be a predictor of mortality.

### Mid- and long-term survival

**Figure 3:**
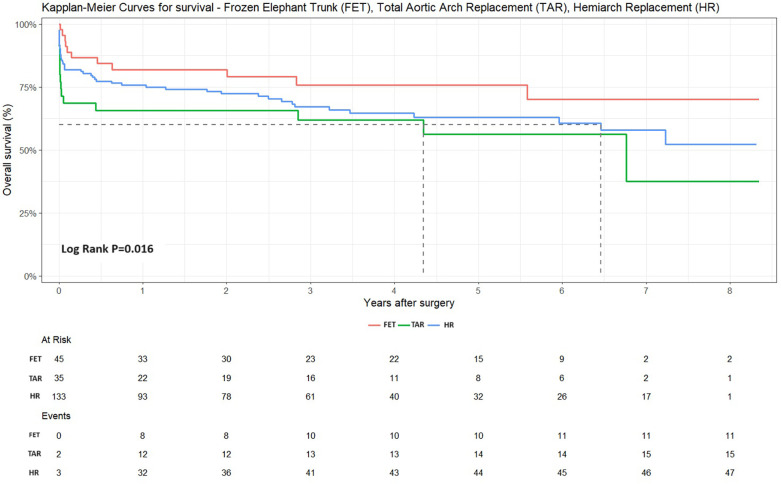
Kaplan–Meier curves of all three patient cohorts: FET, TAR, and HR. The FET technique led to highly reduced mortality rates (Log-rank *P* = 0.016).

After a mean follow-up of 3 ± 2.6 years (FET 3 ± 2.57 years, TAR 3 ± 2.62 years, HR 3 ± 2.53 years), the mid-term mortality rate of discharged patients was 19.4% (34 deaths: 7 FET, 4 TAR, and 23 HR) and the overall mortality rate was 33.8% (72 deaths), elaborated as follows: 11 FET (24.4%), 15 TAR (45.4%), and 46 HR (34.1%) (deaths and percentage of the respective subgroups). Kaplan–Meier curves for survival are shown in Figure 3.

### Reinterventions

A total of eight FET patients (17.8%) received additional endovascular treatment in the descending aorta [seven had true lumen collapse with consequent malperfusion, one of them with an additional dissection of the common carotid artery with a perfusion deficit in the left hemisphere and received stenting, and another patient developed stent graft–induced new entry (SINE)] and only in one patient was open surgical repair needed.

Overall, 13 patients required a reoperation on the ascending aorta or aortic arch, 2 after total aortic arch replacement (6%) due to pseudoaneurysms and 11 after hemiarch replacement (8.1%) due to the development of postdissection aneurysms or anastomosis insufficiency.

## Discussion

Acute aortic dissection remains a serious emergency condition in the field of cardiovascular medicine and a challenge for cardiothoracic surgeons. In addition, intense debates in conferences and the literature regarding the extent of necessary surgical resections often lead to dilemmas in the operating theater.

The standard approach for treating acute aortic dissection is the supracoronary replacement of the ascending aorta up to the proximal aortic arch. Inarguably, this technique is feasible for those with less complex conditions and can be performed with acceptable results. Some techniques can enable a slightly larger resection of dissected tissue (10). However, hemiarch replacement should be performed in standard fashion with open anastomosis. When the entry tear extends further in the aortic arch, in the downstream of the aorta, or even provides multiple entries, a more extensive repair should be done. The highest priority in choosing the surgical strategy is to achieve the best possible outcome for the patient.

The introduction of the frozen elephant trunk technique with prosthesis such as Thoraflex™ Hybrid has simplified the process of extended resections and offers a stabilization of the downstream aorta. It provides a superior hemostasis of the suture line and supports the distal false lumen obliteration with its positive remodeling. This technique of treating false lumen thrombosis reduces late mortality and aortic reintervention rates in the distal thoracic aorta. FET also provides a possible distal landing zone for future endovascular interventions (11).

A previously published institutional experience with the frozen elephant trunk technique in our center has already demonstrated its effectiveness in the surgical treatment of different pathologies, with favorable outcomes (9). These findings are not different from those in the present analysis with even longer follow-up times, which is remarkable considering the serious nature of the disease condition and the treatment emergency. The presented cohort showed a high-risk profile, with many patients being unstable or unconscious at the moment of admission, with signs of cerebral malperfusion.

Despite these clinical features, patients treated with the Thoraflex™ Hybrid prosthesis had a hospital survival rate of almost 90% and a mean follow-up after 3 years of 75%, which was substantially better than the TAR and HR survival rates of 54% and 65%, respectively. Our institutional findings differed in some respects with known aortic registry data (12). In our experience, total aortic arch replacement with FET did not increase the risk of in-hospital mortality. This was even more surprising due to a retrospective calculation of the GERAADA score (which was higher in the FET group, with 24.8%, and lower in the TAR and HR groups, with 20.3% and 21.8%, respectively). A preoperative application of the predicted 30-day mortality score would probably influence the surgical strategy.

Our surgical technique of FET (among others, selective antegrade cerebral perfusion through right subclavian artery cannulation, moderate hypothermia, the standardized perioperative management) produced outcomes comparable to or even superior to those of other high-volume centers (3, 4, 13–16).

Postoperative stroke occurred in 27 patients (12.7%) in the whole cohort, with relatively similar distribution within all subgroups (11.1%, 15.1%, and 12.6% in FET, TAR, and HR, respectively).

The cannulation technique has evolved over the course of time with our experience, and currently, all patients receive an arterial cannulation directly through the right axillary artery. Based on our experience, we recommend an extrathoracic antegrade arterial cannulation via the right axillary artery. This facilitates optimal perfusion of the cerebral vessels and SACP, providing for surgical freedom in aortic arch repair. This type of arterial cannulation has helped improve neurologic results by reducing cerebral complications and, going by our experience, is superior to direct aortic cannulation (17, 18).

Despite the withdrawal of prophylactic preoperative spinal cord protection, we recorded a surprisingly low percentage of paraplegic complications (2.3%, five patients, of whom four received HR). However, there is a lack of sufficient studies on prophylactic cerebrospinal fluid (CSF) drainage (2) to recommend its use.

Current recommendations from the European Association for Cardio-Thoracic Surgery and the European Society for Vascular Surgery advocate “proximalization” of the distal anastomosis from zone 3 to zone 2 (2). The deployment of a stent graft in zone 3 may be associated with a higher rate of laryngeal nerve or spinal cord injury. In our cohort, FET patients suffered more frequently from such complications than other patients (five cases, 11.1% of recurrent nerve palsy). Therefore, “proximalization” of the distal anastomosis should be definitely considered (19).

A systematic review of FET highlighted the feasibility of the technique in elderly and comorbid patients. Despite these findings, indications for treatment in this high-risk group should be clearly defined. In select patients, alternative techniques such as hybrid type II repair provide secure repair with endovascular stenting in zone 0 (20).

In medical practice, FET occupies an indisputable place. However, in this study, a novel device, known as an “Ascyrus Medical Dissection Stent” (AMDS), was introduced, providing a promising and valuable upgrade to hemiarch replacement. It was reported that an AMDS may positively influence aortic remodeling and may be an effective treatment for malperfusion. More data about hemiarch replacement, coupled with additional AMDS implantation information, are definitely required. The AMDS may represent a viable alternative for the management of acute type A dissection, compared with FET, but a careful preoperative evaluation of the patient's individual aortic anatomy regarding potential contraindications has to be undertaken (21).

A proper use of the FET technique guarantees a relative freedom of reoperation, based on our experience. From all 13 patients who required reoperation, none received the Thoraflex™ Hybrid prosthesis during the performance of the primary procedure (11 cases after HR). However, the results can be biased by surgeons’ experience.

Half of re-exploration procedures due to bleeding in the FET group of patients were conducted in the first two years of practice. A learning curve in FET surgery is clearly observable with declining re-exploration rates.

Patients from the hemiarch replacement group were significantly older than those from the other subgroups. It cannot be ruled out that age alone was a limiting factor of repair. We are aware that such an important surgery in elderly patients, particularly in octogenarians, is associated with extremely negative results (22, 23). Surgeons may try to perform restrictive repair to reduce CPB time and to increase the chances of survival in the early postoperative course in the older population. Whether in every single case in this study this course of action was undertaken is doubtful, and therefore, the conclusion cannot be generalized. It is also difficult to assess the results and reflect on them. It should be noted that the other groups also reported similar trends (12, 23, 24).

The gender distribution pattern in the hemiarch group is surprisingly balanced (*P* = 0.006). On the other hand, having a predominantly masculine population of acute dissection patients allows us to consider whether other factors could have influenced the choice of surgical strategy in this cohort. Recent studies reported a significant level of undertreatment of the aortic root and of the aortic arch in women with similar mortality rates and postoperative complications for AAD (23–26).

The duration of surgery and CPB times were shorter in the HR group of patients, as it represents a less complicated repair, than in the Total Arch Replacement group of patients, even though the mortality rates were not predominantly high in these groups.

For patients suffering from a high-risk event such an acute dissection, the frozen elephant trunk technique with the Thoraflex Hybrid prosthesis assures a successful surgical outcome.

The FET technique also impacts the downstream aorta with positive effects on aortic remodeling, leading to a significant increase in true lumen volume and stabilizing false lumen volume, as reported in other studies (8, 27). After the performance of the CET and HR procedures, false lumen volume significantly increased at mid-term follow-up. Due to this increase, the reintervention rate after CET or HR was also higher (28, 29). FET promotes rapid thrombosis of the false lumen and beneficial remodeling of the distal aorta, enabling the performance of procedures in the downstream of the aorta.

Our institutional findings with FET demonstrate promising results as a highly effective operative technique for type A aortic dissection.

## Limitations

A few limitations of this study should be outlined. First, it is retrospective by nature and limited to our institutional experience. Second, our results with regard to low mortality and neurological deficits in the FET group might be biased. This is due to the fact that only one surgeon has specialized in this method and performs Thoraflex™ Hybrid implantations. The diverse experience of other surgeons in the management of such high-risk patients may also have an impact on the results in the TAR and HR groups.

## Data Availability

The raw data supporting the conclusions of this article will be made available by the authors without undue reservation.
